# Alterations of gut microbiota in a mouse model with partial small intestinal obstruction

**DOI:** 10.3389/fmicb.2023.1242650

**Published:** 2023-09-28

**Authors:** Yong Wang, Minzhong Zhang, Lu Jiang, Yiming Gong, Keqiang Liu, Tian Zhang

**Affiliations:** ^1^Department of Pediatric Surgery, the Third Affiliated Hospital of Guangzhou Medical University, Guangzhou, China; ^2^Guangdong Provincial Key Laboratory of Major Obstetric Diseases, Guangzhou, China; ^3^Guangdong Provincial Clinical Research Center for Obstetrics and Gynecology, Guangzhou, China; ^4^Guangdong-Hong Kong-Macao Greater Bay Area Higher Education Joint Laboratory of Maternal-Fetal Medicine, Guangzhou, China; ^5^Department of Pediatric Surgery, Xinhua Hospital Affiliated to Shanghai Jiao Tong University School of Medicine, Shanghai, China; ^6^Shanghai Key Laboratory of Pediatric Gastroenterology and Nutrition, Shanghai, China

**Keywords:** pediatrics, partial small intestinal obstruction, gut microbiota, depleted diversity, intestinal fibrosis

## Abstract

**Introduction:**

Changes in the gut microbiota of patients with partial small intestinal obstruction (PSIO) have not been widely clarified. We aimed to explore bacterial diversity in a PSIO mouse model.

**Methods:**

A PSIO mouse model was established using male C57BL/6 mice, and feces samples from the distal ileum and ileum epithelium tissues were collected. MiSeq sequencing of the 16S rRNA gene was conducted to characterize microbiota diversity and composition. RNA sequencing for differences in transcriptomic programming of the ileum tissue was performed between the PSIO and (Control) Ctrl groups.

**Results:**

Bacterial diversity in the PSIO group was significantly lower than that in the controls. Pseudomonadota was predominant in the feces of the PSIO group. *Unclassified_Muribaculaceae* (*p* = 0.008) and *Akkermansia* (*p* = 0.007) were more abundant in the Ctrl group than those in the PSIO group. Furthermore, *Escherichia_Shigella* (*p* = 0.008) was more predominant in the feces of the PSIO group. The Kyoto Encyclopedia of Genes and Genomes pathways related to metabolism were depleted in the PSIO group. Pathways associated with intestinal fibrosis, including extracellular matrix-receptor interaction, focal adhesion, phosphoinositide 3-kinase (PI3K)-Akt signaling pathway and transforming growth factor (TGF)-beta signaling pathway, which were enriched in ileum epithelial tissue in the PSIO group.

**Conclusion:**

PSIO can lead to changes in the predominant intestinal bacterial groups. Depleted functional profiles of the gut microbiota were identified in the PSIO group. Functional pathways associated with intestinal fibrosis were activated by PSIO. The potential regulation by the microbiota needs to be explored in the future.

## Introduction

Partial small intestinal obstruction (PSIO) is defined as a partial blockage of the small bowel, which prevents contents in the lumen from moving through the gastrointestinal tract (Chen et al., 2006). Congenital or acquired etiologies can lead to small intestinal obstruction in children, such as necrotic enterocolitis (NEC), post-surgical adhesions, and intestinal atresia. Intestinal ischemia and preformation are usually caused by complete obstruction due to sharp distention of the intestinal wall. Unlike complete intestinal obstruction, PSIO does not cause severe symptoms and is often overlooked. However, PSIO can result in poor prognosis without proper treatment (Milad et al., 2007). Surgical intervention is necessary in cases of intestinal strangulation or complete intestinal obstruction (Kendrick, 2009). Patients with PSIO may experience a high complication rate, with up to 30% of them experiencing strangulation. As PSIO progresses, luminal occlusion results in fluid accumulation and gas production from bacterial overgrowth proximal to the obstruction site. The intestinal wall gradually becomes distended, thickened, and finally paralyzed due to blockage, which can usually result in complications such as enteritis, sepsis, and even complete intestinal obstruction. Studies focusing on the mechanism of PSIO-induced pathophysiological changes are rare. In addition, the influence of PSIO on immunity, metabolism, and other physical activities is yet to be explored.

Gut functions are profoundly influenced by the gut microbiota. A significant decrease in the diversity of the gut microbiota and alteration of the function of these bacteria, known as gut dysbiosis, is strongly correlated with physiological dysfunction, metabolic disturbances, impaired nutrient absorption, altered metabolic activities, and compromised immune regulation within the gastrointestinal tract (GI). Recently, there has been increased exploration into the role of gut dysbiosis in gastrointestinal motility disorders. The interaction between the enteric nervous system (ENS) and gut microorganisms, including bacteria, viruses, and parasites, has been documented (Holland et al., 2021). Changes in gut microbiota may play a vital role in the pathogenesis of PSIO. In a mouse model with partial colonic obstruction, the diversity of microbiota reportedly increases, and is associated with a decrease in Bacillota and increase in Pseudomonadota and Bacteroidota (Hegde et al., 2018). Furthermore, complete small bowel obstruction leads to a decrease in Bacillota, increase in Pseudomonadota, Verrucomicrobia, and Bacteroidota, and disruption of the intestinal mucosal barrier (Mo et al., 2021). Currently, clinical studies investigating alterations in intestinal microbiota among patients with PSIO are limited. Certain GI motility disorders, such as chronic intestinal pseudo-obstruction (CIPO) and Hirschsprung’s disease, can manifest with symptoms resembling PSIO. Small intestinal bacterial overgrowth (SIBO) resulting from gut dysbiosis in GI dysmotility is prevalent in these patients, characterized by an overabundance of opportunistic pathogens like *Streptococcus* and *Escherichia coli*, coupled with a decrease in Bacillota and *Bifidobacterium* (Bures et al., 2010; Chantakhow et al., 2021). Moreover, SIBO contributes to increased gut bacterial translocation and immunodeficiency, potentially underpinning subsequent systemic inflammatory responses, sepsis, and the development of multiple organ dysfunction syndrome (MODS). Therefore, maintaining the balance of gut microbiota has emerged as a therapeutic avenue for individuals with GI dysmotility (Zhang et al., 2022). Gu et al. demonstrated that fecal microbiota transplantation (FMT) can alleviate symptoms in CIPO patients (Gu et al., 2017).

In this study, we aimed to explore the alterations of microbiota in an animal model of PSIO, to gain insight into the mechanism of PSIO-induced pathophysiological changes. Our study reveals prominent alterations in gut microbial diversity within PSIO mice, characterized by a reduction in beneficial bacterial populations and a concurrent rise in opportunistic pathogens. Furthermore, we observed a diminished metabolic capacity of the fecal microbiota attributed to the presence of PSIO. Additionally, we noted an enrichment of pathways related to intestinal fibrosis within the intestinal epithelium of PSIO mice. Our findings suggest that clarifying the composition and function of the gut microbiota in PSIO may help to better understand the mechanism of PSIO-associated complications, which might be a potential therapeutic strategy to maintain intestinal and systemic homeostasis in such patients.

## Methods and materials

### Animal model

Twelve male C57BL/6 mice aged 8 weeks were divided into two groups: Control (Ctrl, *n* = 4) and PSIO (*n* = 8). All mice underwent surgery for partial intestinal obstruction as previously described (Georgopoulos et al., 2020). Anesthesia was induced with an intraperitoneal ketamine injection (70 mg/kg, Xylapan, Vetoquinol, France), and the skin was cleaned with 70% ethanol. A 3.0 cm abdominal incision was made to expose the intraperitoneal cavity. The cecum was carefully identified and resected. An incision was made in the mesentery parallel to and below the ileum and proximal to the colon. An autoclaved silicone ring (6 mm in length, 4 mm exterior diameter, and 3.5 mm interior diameter) was prepared and cut longitudinally to open the tubing. Subsequently, one end of the opened ring was inserted through the incision and brought into contact with the other end. The ring was closed with sutures encasing the ileum within the ring. Finally, the intestine was placed back into the intraperitoneal cavity and the abdominal cavity was closed with sutures. For mice in the Ctrl group, an incision was made in the midline and subsequently closed. After two weeks of feeding, the feces of the mice were collected for further examination.

### Fecal samples collection

One fecal sample was collected from each subject. All samples were collected immediately into a sterile tube after defecation and stored at −20°C. Thereafter, it was stored at −80°C for 24 h for DNA extraction.

### Bacterial DNA extraction and polymerase chain reaction amplification from stool samples

A QIAamp Fast DNA Stool Mini Kit (Qiagen, Hilden, Germany) was used to extract microbial DNA. The concentration of the extracted DNA was measured using a Nano-Drop 2000 spectrophotometer (Thermo Scientific, Waltham, MA, United States). PCR amplification of the 16S rRNA genes was performed (Dennis et al., 2013) using PCR primers specific for the 515–806 (V3–V4) regions. The PCR assays were performed in triplicate with the following mixture: 20 mL reaction solution with 10 ng of template DNA, 4 μL of PCR reaction buffer, 0.4 mM of each primer, 2.5 mM of deoxyribonucleotide triphosphate (dNTPs), and 0.5 U of TransStartFastPfu DNA polymerase (TransGen Biotech, Beijing, China). PCR analysis was performed under the following conditions: 95°C for 4 min, followed by 27 cycles of 95°C for 30 s, 55°C for 30 s, 72°C for 45 s, and a final analysis at 72°C for 10 min.

### Illumina MiSeq sequencing

DNA amplicons were collected from 2% agarose gels, purified using the AxyPrep DNA Gel Extraction Kit (Axygen Biosciences, Union City, CA, United States), and quantified using QuantiFluo-ST (Promega, Madison, WI, United States), according to the manufacturer’s instructions. The purified amplicons were pooled in equimolar and paired-end sequences (2 × 250) on an Illumina MiSeq platform.

### Processing of sequencing data

Raw Fastq files were demultiplexed and quality-filtered using QIIME (version 1.17) with the following criteria: (1) 300 bp reads were truncated at any site receiving an average quality score of <20 over a 50 bp sliding window, deleting the truncated reads that were < 50 bp; (2) exact barcode matching, two nucleotide mismatches in primer matching, and reads containing ambiguous characters were removed; and (3) only sequences overlapping >10 bp were assembled according to their overlap sequence. Reads that could not be assembled were excluded. Operational taxonomic units (OTUs) were clustered with a 97% similarity cut-off using UPARSE (version 7.1), and chimeric sequences were identified and discarded using UCHIME (version 4.2). The phylogenetic affiliation of each 16S rRNA gene sequence was analyzed using the RDP Classifier against the SILVA (SSU117/119) 16S rRNA database.

### RNA sequencing of the intestinal epithelium

Fresh ileum tissues were collected and stored at −80°C for further examination. Total RNA was extracted from ileum tissue samples according to the manufacturer’s instructions (Invitrogen, Carlsbad, CA, United States). RNA quality was determined using a 2,100 Bioanalyzer (Agilent, Stanta Clara, CA, United States) and quantified using an Nano-Drop 2000. An RNA-sequence transcriptome library using RNA samples with high quality was prepared using the TruSeq™ RNA sample preparation Kit (Illumina, San Diego, CA, United States). The paired-end RNA library was sequenced using HiSeq xten/NovaSeq 6,000 sequencer (Illumina, San Diego, CA, United States).

### Function prediction

Bacterial functions were predicted using the Phylogenetic Investigation of Communities by Reconstruction of Unobserved States (PICRUSt) package. The Operational taxonomic unit (OUT) was selected against the Greengenes database (version 13.5) using Mothur. A compatible biological observation matrix (BIOM) table was generated using the PICRUSt program. The metagenome was predicted was using the OTU table after 16S rRNA copy number normalization. The predicted functional genes were annotated using Kyoto Encyclopedia of Genes and Genomes (KEGG) analysis.

### Statistics

Statistical differences between the PSIO and Ctrl groups were analyzed by performing a two-tailed Wilcoxon test using SPSS (version 20.0). Data are presented as the mean ± SD. Statistical significance was set at *p* < 0.05.

## Results

### Model

**Figure 1:**
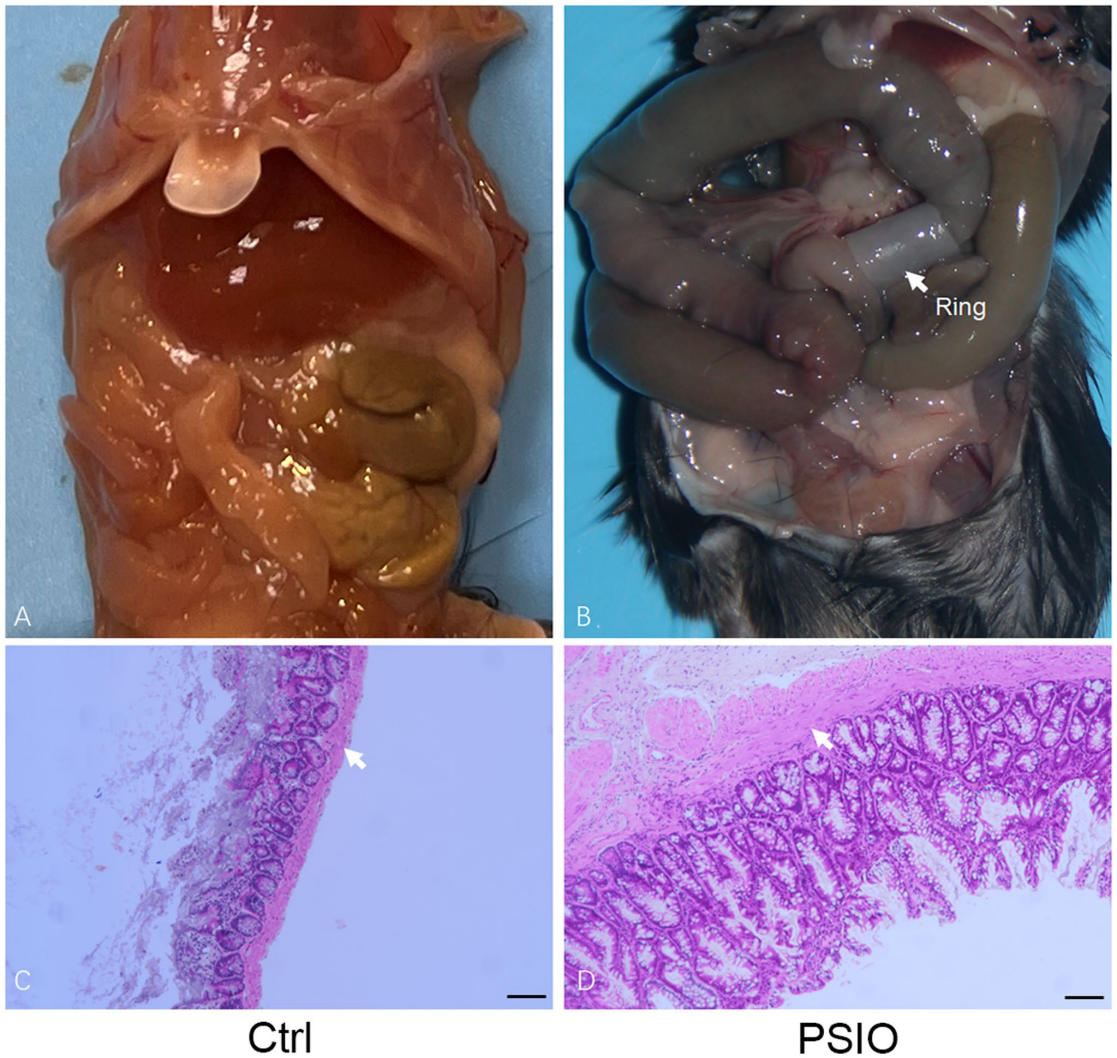
Partial intestinal obstruction surgically induced in a mouse model. **(A)** Gross images of the gastrointestinal tract in mice that surgery. **(B)** Gastrointestinal tract dissected from mice. White arrows point to the smooth muscle layer. Ctrl, control; PSIO, partial small intestinal obstruction.

Mice in the PSIO group showed abdominal distension during feeding, accompanied by anorexia and weight loss. After the mice were euthanized, we observed significant dilation of the small intestine proximal to the ligation and decreased content in the distal small intestine and colon. The proximal ileum was stained with hematoxylin & eosin (H&E); the ileal smooth muscles of the mice in the PSIO group were hypertrophied. The above results show that the model can effectively simulate the morphological changes in PSIO (Figure 1).

### Bacterial diversity between groups

**Figure 2:**
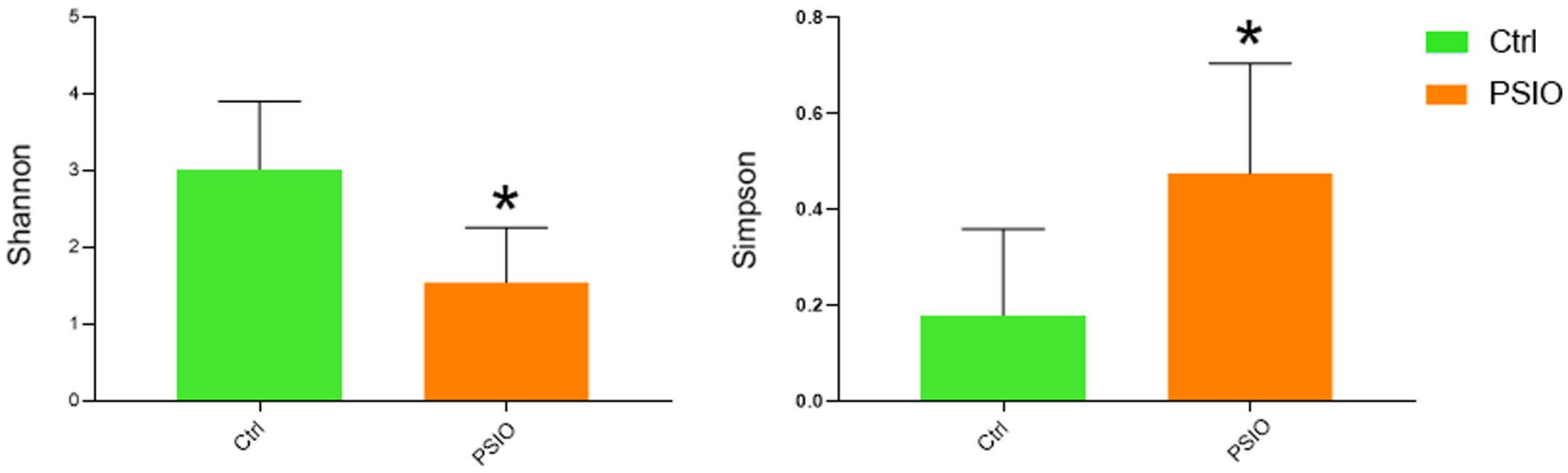
Microbial alpha diversity among between Ctrl and PSIO group. The Wilcoxon sum-rank test analyzed differences between the two groups. *Significant difference *p* < 0.05. Ctrl, control; PSIO, partial small intestinal obstruction.

**Figure 3:**
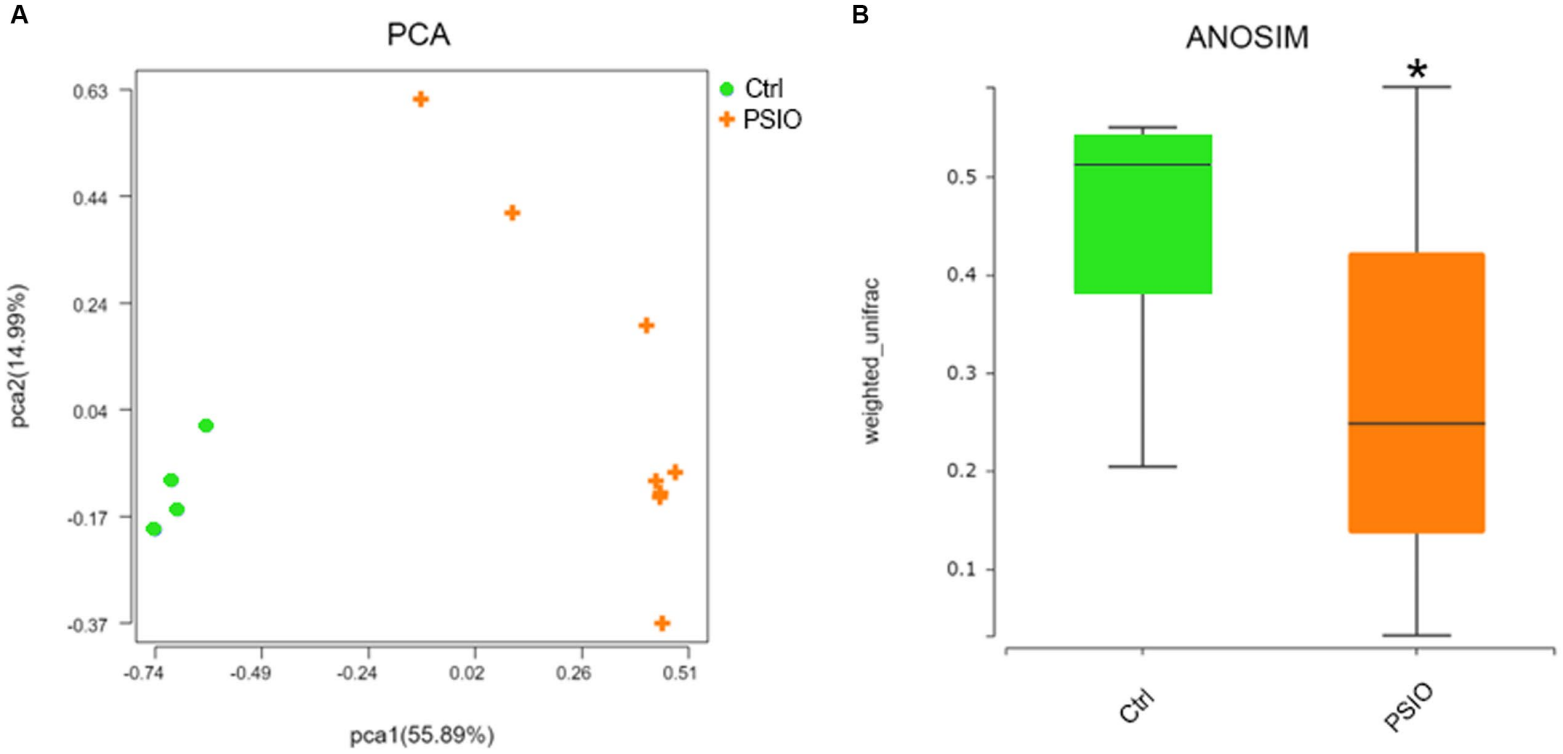
Fecal bacterial beta diversity in feces of controls and PSIO mice. **(A)** PCA with Bray–Curtis distance matrix. **(B)** Significant inter-group difference detected by ANOSIM analysis in Bray–Curtis distance. PCA, principal component analysis; ANOSIM, analysis of similarities. Ctrl, control. PSIO, partial small intestinal obstruction. ANOSIM was performed with Wilcoxon sum-rank analysis.

A total of 464 OTUs were identified at 97% similarity level in all fecal samples. Good’s coverage was >99% in each group. We compared the alpha diversity, which was presented using the Shannon and Simpson index, in the feces of mice in the PSIO and Ctrl groups. Mice in PSIO group owned lower Shannon index and higher Simpson index, meaning a lower microbial diversity than that in the Ctrl group (Figure 2). The gut microbiota was analyzed and compared with the relative abundance of OTUs using the Bray–Curtis distance matrix for beta diversity analysis. Principal component analysis (PCA) revealed dissimilarities in microbial compositions between the two groups, and samples from each group clustered together. Analysis of similarities (ANOSIM) showed significant differences between the groups, which suggested that the separation between the two groups was good, and intergroup variations were significantly greater than intragroup variations (Figure 3).

### Bacterial composition differences at the phylum level

**Figure 4:**
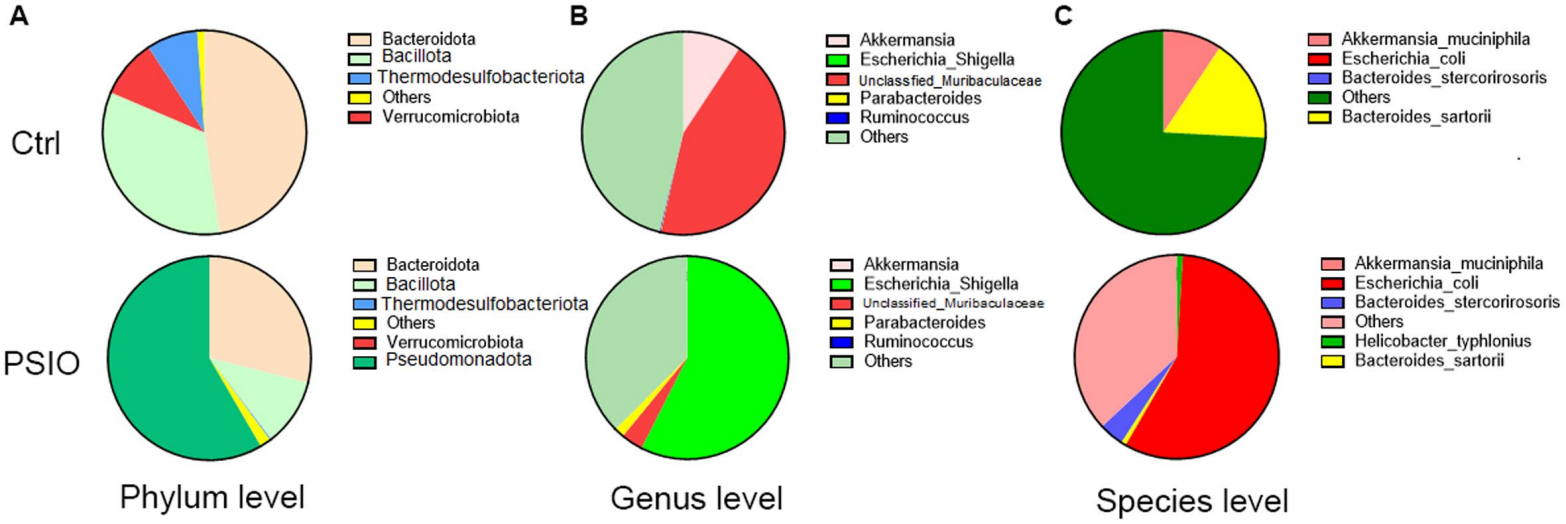
Pie charts representing the overall microbial composition of feces obtained from the PSIO group and controls. **(A)** At the phylum level. **(B)** At the genus level. **(C)** At the species level. Ctrl, control; PSIO, partial small intestinal obstruction.

The dominant phyla in the two groups were Bacteroidota, Bacillota, and Pseudomonadota; however, the proportions were different (Figure 4). Compared to the Ctrl group, mice in the PSIO group had higher levels of Pseudomonadota (58.41% ± 31.49% vs. 0.78 ± 0.88%, *p* = 0.008) and lower levels of Verrucomicrobiota (0.02% ± 0.33% vs. 9.33 ± 4.29%, *p* = 0.007) and Bacillota (10.70 ± 14.11% vs. 33.81 ± 24.93%, *p* = 0.075).

### Bacterial composition differences at the genus level

The bacterial composition of the two groups at the genus level is shown in Figure 4. Genus-level bacterial taxa distinguishing the feces of the Ctrl and PSIO groups included *unclassfied_Muribaculaceae* (genera of Muribaculaceae, 44.25 ± 32.26% vs. 3.51 ± 2.58%, *p* = 0.008) and *Akkermansia* (genus of Akkermansiaceae, 9.33 ± 4.29% vs. 0.02 ± 0.03%, *p* = 0.007), which were more abundant in Ctrl group but depleted in mice with intestinal obstruction, suggesting that these genera were indicative of a relatively normal state. In contrast, *Escherichia_Shigella* was dominant in the PSIO group (57.44 ± 31.64% vs. 0.00 ± 0.00%, *p* = 0.008).

### Bacterial composition differences at the species level

The bacterial compositions of the two groups at the species level are shown in Figure 4. Mice with PSIO had a higher abundance of *Escherichia coli* (57.38 vs. 31.62% vs. 0.00 ± 0.00%, *p* = 0.008) and lower levels of *Akkermansia muciniphila* (0.02 ± 0.03% vs. 9.33 ± 4.29%, *p* = 0.007) at the species level than the Ctrl group did.

### Functional analysis of gut microbiota

**Figure 5:**
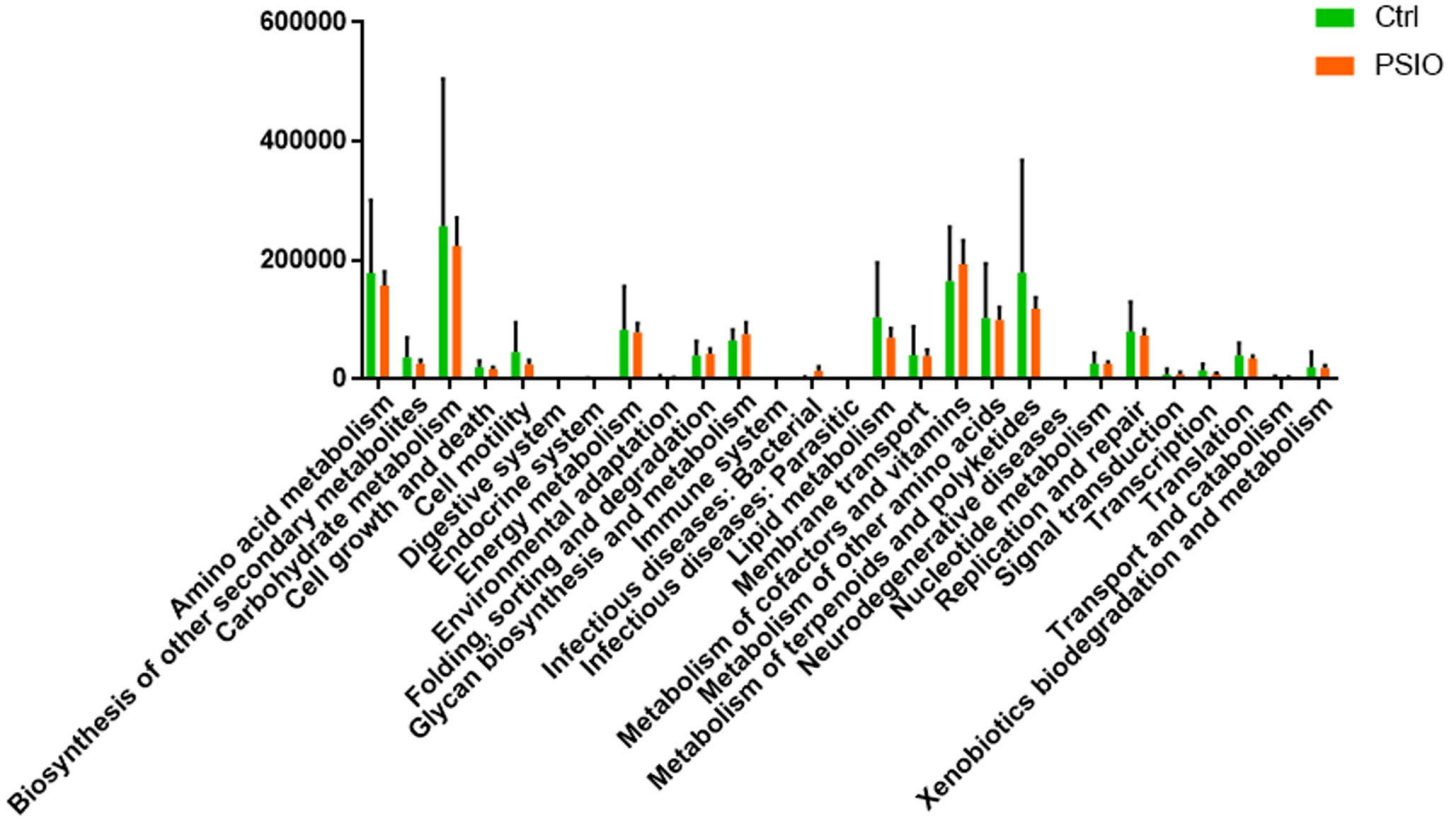
KEGG pathways exhibit between-group differences in relative abundance. KEGG, kyoto encyclopedia of genes, and genomes. Ctrl, control; PSIO, partial small intestinal obstruction.

We identified differences in the functional microbial pathways between mice in the PSIO and Ctrl groups, based on 16S rRNA sequencing data. At KEGG level 2, mice in the PSIO group had activation of more functions involved in infectious diseases than the Ctrl group did. Furthermore, the levels of functions responsible for lipid metabolism, transcription, and metabolism of secondary metabolites were depleted in the PSIO group (Figure 5).

### Difference of transcriptomic programming of intestinal epithelium

**Figure 6:**
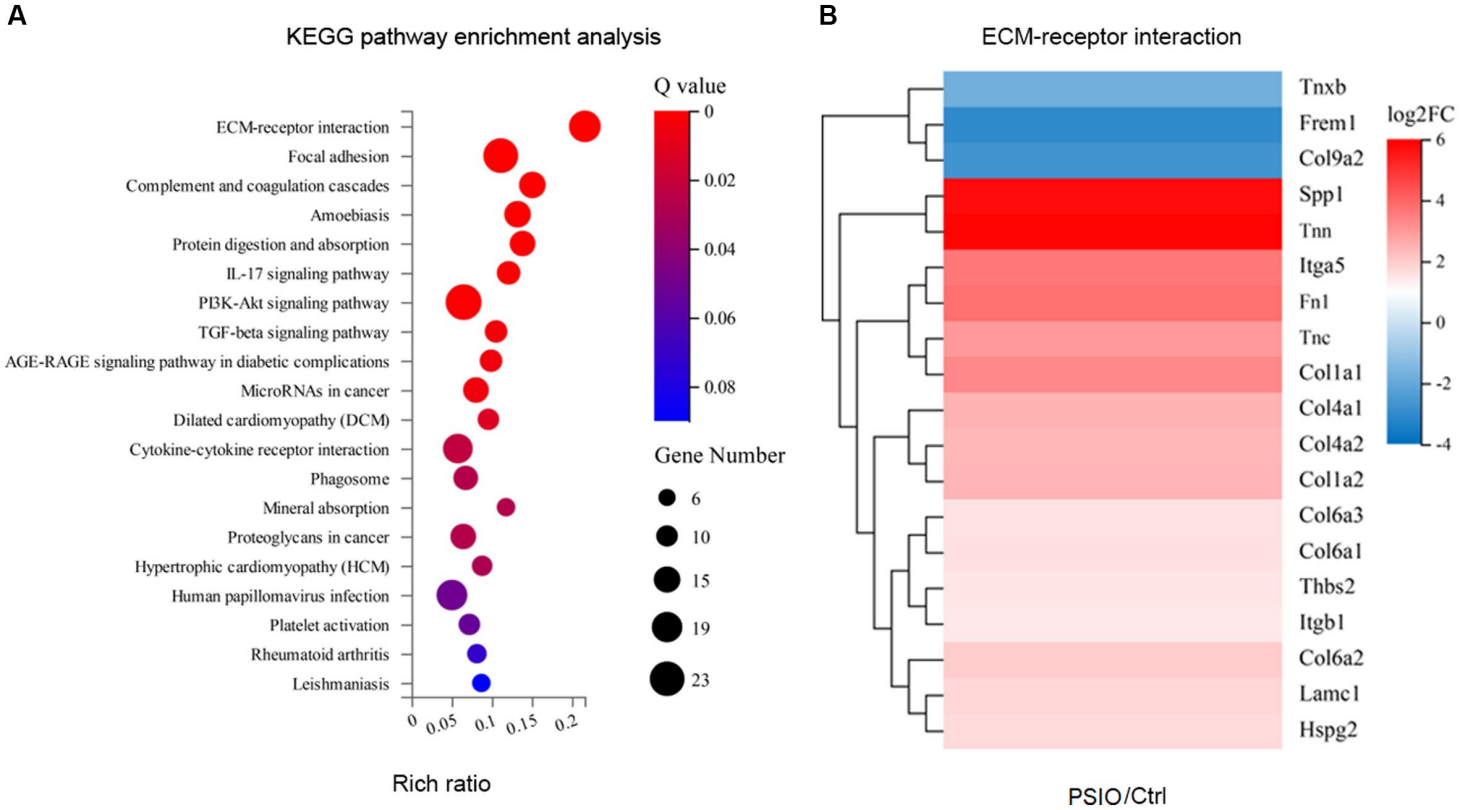
KEGG enrichment analyses of differences in the transcriptomes of the ileal tissue of mice between the groups. **(A)** Heat map of pathways. **(B)** Differences of gene expressions of ECM-receptor interaction pathway. KEGG, kyoto encyclopedia of genes and genomes; ECM, extracellular matrix. Ctrl, control; PSIO, partial small intestinal obstruction.

We analyzed the differences in the transcriptomes of the ileal tissue of mice between the groups. Compared to the Ctrl group, mice with PSIO demonstrated an evidently different transcriptome, with 271 significantly differentially expressed genes (DEGs), among which 72.3% were unregulated. KEGG enrichment analyses further demonstrated that the intestinal epithelium was enriched in pathways associated with intestinal fibrosis, including extracellular matrix (ECM)-receptor interaction, focal adhesion, phosphoinositide 3-kinase (PI3K)-Akt signaling pathway, and transforming growth factor (TGF)-beta signaling pathway. ECM-receptor interaction was the leading upregulated pathway, accompanied by high expression of Spp1, Tnn, Itga5, Tnc, Fn1, Col1a, and Col4a. Intestinal tissue collected from mice in the PSIO group was also enriched with transcripts of intestinal immunology, including complement and coagulation cascades and the interleukin (IL)-17 signaling pathway. Additionally, other pathways such as microRNAs in cancer, cytokine-cytokine receptor interactions, and phagosomes were also upregulated by PSIO (Figure 6).

## Discussion

PSIO increases complications such as enteritis, sepsis, and multiple MODS, resulting in poor diagnosis and high medical cost. However, the mechanisms of PSIO-induced complications remain unexplained. Gut dysbiosis has strong correlation with GI diseases. Studies focusing on PSIO are still rare. Patients with CIPO can present with symptoms of PSIO and gut dysbiosis is common in such patients, which influences the gut motility, ENS and intestinal endocrine system (IES) of GI (Radocchia et al., 2021). This study provides new insights into the gut microbial distribution in a PSIO mouse model compared with controls. The results showed that the microbial diversity of mice with PSIO was lower than that of the Ctrl group, which was relatively similar to some reports focusing on the gut microbiota in intestinal obstruction. In mice with PSIO, higher levels of Pseudomonadota and Bacteroidota were observed, accompanied by decreased levels of Bacillota. Pseudomonadota, whose members are mainly facultative or obligate, account for only a small proportion of the gut microbiota in healthy people (Sun et al., 2021).

The overgrowth of Pseudomonadota is reportedly caused by gastrointestinal environmental changes, such as lumen pH value (Duncan et al., 2009) and intestinal structure (Zhang et al., 2020). In patients with PSIO, the permeability of the intestinal epithelial barrier and changes in gut motility may result in changes in lumen pH value. In addition, children with PSIO may require parenteral nutrition support (Andersen et al., 2020), which is one reason for the overabundance of Pseudomonadota. Children with PSIO are at a high risk of intestinal-derived sepsis (Vaishnavi, 2013). There is evidence that overgrowth of Pseudomonadota is strongly related to high-level intestinal inflammation and increased epithelial permeability (Shin et al., 2015).

*Unclassfied_Muribaculaceae* and *Akkermansia* were significantly decreased in mice with PSIO. *Akkermansia* resides in the human gut and its effects on human metabolism have been studied. One *Akkermasia* species, named *Akkermansia muciniphila*, reportedly reduces obesity, diabetes, and inflammation (Gomes et al., 2018). In addition, high levels of gut *Akkermansia muciniphila* are associated with an increased risk of type 2 diabetes and obesity as reported (Hasani et al., 2021). *Unclassfied_Muribaculaceae* and Muribaculaceae was mainly studied in a mouse model, and its abundance was strongly correlated with short-chain fatty acid (SCFA) concentrations of enteric lumen (Smith et al., 2021). SCFA are the predominant metabolites of the gut microbiota (Morrison and Preston, 2016). SCFA showed an anti-inflammatory effect in the gut by regulating the function of regulatory T cells. In our study, *Escherichia*, which includes several commensal members in the human gut, was sharply increased in the feces of mice with PSIO. This phenomenon was similar to that observed in other studies focusing on the gut microbiota and intestinal obstruction (Generoso et al., 2011).

*Escherichia* has a strong relationship with the microbial balance in the human gut (Chaudhuri and Henderson, 2012; Guglietta, 2017). Certain strains of *Escherichia*, most notably the serotypes of *Escherichia coli*, are human pathogens and correlate with gastrointestinal diseases, ranging from simple diarrhea to dysentery-like conditions, urinary tract infections, and a wide range of other pathogenic states (Chaudhuri and Henderson, 2012). *Escherichia coli* and its overgrowth can induce a higher level of lumen inflammation (Palmela et al., 2018). In contrast, increased epithelial barrier permeability is partly caused by intestinal inflammation and the increased translocation of *Escherichia coli* (Lamb-Rosteski et al., 2008). Mitrea et al. reported the inhibition of *Escherichia coli* by *Lactobacillus Plantarum* (Mitrea et al., 2017). Probiotics are well known about their effect on antimicrobial action and immunomodulatory activity. There is a link between gut dysbiosis and metabolic endotoxemia, which can occur on patients with intestinal obstruction. Matei-Latiu et al. proved that treatment with probiotics based on *Bacillus subtilis*, *Bacillus licheniformis,* and *Pediococcus acidilactici* strains resulted in a notable improvement in the general health status of the dogs with gut dysbiosis (Matei-Latiu et al., 2021). These findings might suggest that an imbalance in microbial diversity plays an important role in the pathophysiology of PSIO and regulation of gut microbiota in PSIO might be a potential therapy in future.

In the current study, a depleted carbohydrate metabolism pathway module was found in mice with PSIO compared to that in the Ctrl group at KEGG level 2. In our study, the level of Enterobacteriaceae increased, which might be related to dysfunction of carbohydrate metabolism. Several of its members use the Entner-Doudoroff pathway for glucose metabolism and thus, may not be able to catabolize it through glycolysis (Zou et al., 2018). In addition, amino metabolism pathway was also interrupted by PSIO. Microbiota in the small bowel is reportedly involved in host metabolism through various physiological processes (Martinez-Guryn et al., 2018). Thus, abnormalities in metabolic processes in PSIO might correlate with changes in the gut microbial composition.

In PSIO intestinal ileum tissue, the expression of pathways related to intestinal fibrosis, including ECM-receptor interaction, focal adhesion, PI3K-Akt signaling, an TGF-β signaling, increased. Intestinal fibrosis is a serious complication of recurrent intestinal inflammation-related tissue injuries, such as inflammatory bowel disease (IBD) (Latella et al., 2015). The mechanism underlying intestinal fibrosis has not been fully elucidated. The activation of immune and mesenchymal cells, such as intestinal myofibroblasts, triggers the secretion of cytokines, proteases, and chemokines, and plays an important role in the activation of intestinal fibrosis (Wang et al., 2021). ECM remodeling is essential for recovery from intestinal damage (Bonnans et al., 2014). However, extensive ECM remodelling is associated with intestinal fibrosis. Qi et al. reported that the gut microbiota derived from major depressive disorder (MDD) patients promotes the expression of cola4a1, a2, lamc3, and flna, which are involved in the ECM-receptor interaction and focal adhesion pathways (Qi et al., 2020). In the dextran sulphate sodium (DSS)-induced chronic colitis model, the PI3K-Akt and TGFβ pathways are upregulated (He et al., 2021). Mishima et al. showed that normal resident microbiota helps maintain hemostasis through the PI3K pathway (Mishima et al., 2019). In addition, Ihara et al. found that the gut microbiota could inhibit intestinal inflammation by modulating TGF-β (Ihara et al., 2016). Thus, the gut microbiota might contribute to pathophysiological changes in PSIO by regulating intestinal fibrosis.

In mice with PSIO, genes associated with intestinal immunity, including the IL-17 pathway and the complement and coagulation cascades, were enriched. IL-17 producing cells are increased in the submucosa and muscularis propria, and are essential for the development of colonic inflammation. Dupraz et al. reported that gut microbiota-derived short-chain fatty acids suppressed intestinal inflammation by regulating IL-17 levels in human and mice models (Dupraz et al., 2021). Meanwhile, the complement and coagulation cascade pathways were induced under conditions of high levels of intestinal oxidative stress and contributed to pro-inflammatory effects (Cheng et al., 2021). The relationship between gut microbiota and the complement and coagulation cascades has not been widely studied. Xue et al. found that oleanolic acid suppressed intestinal inflammation by reshaping the gut microbiota, and the expression of immune-related genes in the complement and coagulation cascade pathways also changed (Xue et al., 2022). In addition, Lin et al. found that gut microbiota might contribute to the maturation of lymphocytes in the gut-associated lymphoid tissues (GALT) and bowel obstruction resulted in lymphocyte generation and maintenance in lymphoid organs, which leaded to immune deficiency (Lin et al., 2022). Thus, in PSIO, gut microbiota may play a vital role in regulating intestinal immunity and the mechanism should be furthered explored.

In the present study, we established a mice PSIO model of PSIO to explore gut microbial changes. This is one of the most cost-effective ways to study PSIO *in vivo*. We used a silicone ring of a specific size to create a partial obstruction, preventing blockage or complete blockage. We observed small intestinal smooth muscle hypertrophy, which mimicked human intestinal obstruction. Clinically, patients with PSIO usually receive systemic treatments, including antibiotics. Thus, it might be difficult to clarify the real changes in gut microbial diversity in these patients. The PSIO mouse model provides a tool for further studies. For instance, the PSIO mouse is an ideal hypertrophy model for intestinal obstruction in which smooth muscle cells, intestinal cells of Cajal, and PDGFAα+ cells are abnormally remodeled during intestinal obstructions (Ha et al., 2018).

This study had a few limitations. Firstly, the sample size was relatively small and only focused on the mouse model. However, patients with PSIO usually receive intravenous antibiotics, which could influence the diversity of the gut microbiota. Therefore, a study with a large human cohort should be performed to provide a comprehensive picture of changes in the gut microbiota in PSIO. Secondly, gut microbiota’s metabolites like SCFAs were not considered for this experimental design. These metabolites are important to be analyzed in close connection with the dominant microbiotain future studies. Meanwhile, the relationship between changes in microbiota and intestinal fibrosis and immunity-related genes, and the mechanism of regulation of these genes remains unclear. This relationship and mechanism should also be explored in the future.

## Conclusion

In summary, marked alterations in the microbial composition of PSIO models and Ctrl mice were demonstrated in this study. An overabundance of Pseudomonadota and *Escherichia coli* and lower levels of *Akkermansia muciniphila* were found in the PSIO mice than in the controls. The metabolic function of the fecal microbiota was depleted in the PSIO group. The PSIO intestinal epithelium was enriched in pathways associated with intestinal fibrosis, including ECM-receptor interaction, focal adhesion, PI3K-Akt signaling pathway, and TGF-beta signaling pathway. Clarifying the composition and function of the gut microbiota in PSIO may help to better understand the mechanism of PSIO-associated complications and maintain intestinal homeostasis in such patients.

## Data availability statement

The data presented in the study are deposited in the China National Center For Bioinformation. The accession number is CRA012605. Please access it from the following link: https://bigd.big.ac.cn/gsa/browse/CRA012605.

## Ethics statement

The animal study was approved by Xin Hua Hospital Animal Use Committee. The study was conducted in accordance with the local legislation and institutional requirements.

## Author contributions

YW and TZ: study conception and design. KL and YG: data acquisition. TZ and MZ: analysis and data interpretation. TZ and YW: drafting of the manuscript. YW and LJ: critical revision and submit this form with the manuscript. All authors contributed to the article and approved the submitted version.

## Funding

This work was supported by Shanghai Sailing Program (20YF1429300, 22YF1436700), National Natural Science Foundation of China (81800448) and Shanghai Science and Technology Innovation Program (22S31904200).

## Conflict of interest

The authors declare that the research was conducted in the absence of any commercial or financial relationships that could be construed as a potential conflict of interest.

## Publisher’s note

All claims expressed in this article are solely those of the authors and do not necessarily represent those of their affiliated organizations, or those of the publisher, the editors and the reviewers. Any product that may be evaluated in this article, or claim that may be made by its manufacturer, is not guaranteed or endorsed by the publisher.
